# Altered Brain Function in Treatment-Resistant and Non-treatment-resistant Depression Patients: A Resting-State Functional Magnetic Resonance Imaging Study

**DOI:** 10.3389/fpsyt.2022.904139

**Published:** 2022-07-22

**Authors:** Jifei Sun, Yue Ma, Limei Chen, Zhi Wang, Chunlei Guo, Yi Luo, Deqiang Gao, Xiaojiao Li, Ke Xu, Yang Hong, Xiaobing Hou, Jing Tian, Xue Yu, Hongxing Wang, Jiliang Fang, Xue Xiao

**Affiliations:** ^1^Department of Radiology, Guang'anmen Hospital, China Academy of Chinese Medical Sciences, Beijing, China; ^2^Department of Psychiatric, Beijing First Hospital of Integrated Chinese and Western Medicine, Beijing, China; ^3^Department of Neurology, Xuanwu Hospital, Capital Medical University, Beijing, China

**Keywords:** treatment-resistant depression, non-treatment-resistant depression, fMRI, amplitude of low-frequency fluctuation, regional homogeneity

## Abstract

**Objective:**

In this study, we used amplitude of low-frequency fluctuation (ALFF) and regional homogeneity (ReHo) to observe differences in local brain functional activity and its characteristics in patients with treatment-resistant depression (TRD) and non-treatment-resistant depression (nTRD), and to explore the correlation between areas of abnormal brain functional activity and clinical symptoms.

**Method:**

Thirty-seven patients with TRD, 36 patients with nTRD, and 35 healthy controls (HCs) were included in resting-state fMRI scans. ALFF and ReHo were used for image analysis and further correlation between abnormal brain regions and clinical symptoms were analyzed.

**Results:**

ANOVA revealed that the significantly different brain regions of ALFF and ReHo among the three groups were mainly concentrated in the frontal and temporal lobes. Compared with the nTRD group, the TRD group had decreased ALFF in the left/right inferior frontal triangular gyrus, left middle temporal gyrus, left cuneus and bilateral posterior lobes of the cerebellum, and increased ALFF in the left middle frontal gyrus and right superior temporal gyrus, and the TRD group had decreased ReHo in the left/right inferior frontal triangular gyrus, left middle temporal gyrus, and increased ReHo in the right superior frontal gyrus. Compared with the HC group, the TRD group had decreased ALFF/ReHo in both the right inferior frontal triangular gyrus and the left middle temporal gyrus. Pearson correlation analysis showed that both ALFF and ReHo values in these abnormal brain regions were positively correlated with HAMD-17 scores (*P* < 0.05).

**Conclusion:**

Although the clinical symptoms were similar in the TRD and nTRD groups, abnormal neurological functional activity were present in some of the same brain regions. Compared with the nTRD group, ALFF and ReHo showed a wider range of brain area alterations and more complex neuropathological mechanisms in the TRD group, especially in the inferior frontal triangular gyrus of the frontal lobe and the middle temporal gyrus of the temporal lobe.

## Introduction

Major depressive disorder (MDD) is a common clinical disorder of the psychiatric system, characterized by significant and persistent depressed mood, reduced interest, reduced cognitive function and somatic disturbances (1). MDD has a high global prevalence, affecting over 300 million people worldwide, and is expected to be the leading global disease burden by the end of 2030 (2). Despite the availability of psychological, physical and pharmacological therapies, 30–40% of people with MDD still fail to respond to these treatments (3). According to the most common previous definition of treatment-resistant depression(TRD), the patient does not improve after at least two or more treatments of adequate dose and duration (4). Therefore, the study of TRD has become a challenging research domain, where substantial effort has been invested worldwide (5).

TRD is a complex disorder whose pathogenesis is not fully understood, and it reflects the coexistence of multiple depressive subtypes, psychiatric comorbidity, and coexisting medical illnesse (6). Compared with the non-treatment-resistant depression (nTRD), patients with TRD are more difficult to treat and consume more treatment costs (4, 7). Overall medical costs for TRD patients are almost twice or more than twice as high as those for nTRD patients (8, 9). In terms of clinical symptoms, compared with nTRD patients, TRD patients had more severe depressive symptoms, cognitive impairment, anxiety and distress, longer onset period, and lower work productivity (7, 10–12). Therefore, it is necessary to explore the biomarkers of TRD, which in turn will facilitate the understanding of the differences in the pathogenesis of TRD and nTRD.

Currently, although brain tissue or cerebrospinal fluid is an ideal resource for studying MDD biomarkers, it is more difficult to obtain in the clinic (13). Although peripheral blood proteins are easy to obtain but there are many types of studies and no uniform standards have been established (14). In recent years, resting-state functional magnetic resonance imaging (rs-fMRI) has been gradually applied in the field of psychiatric disorders, such as MDD (15, 16), schizophrenia (17) and autism (18), because of its simplicity and ease of acquisition. It has also been further applied to the study of subtypes of depression (19–21). The application of rs-fMRI technology facilitates a deeper understanding of the neuropathology of depression and the development of effective antidepressant drugs (22).

Regional homogeneity (ReHo) and amplitude of low-frequency fluctuations (ALFF) are commonly used in rs-fMRI to study functional brain activity. ReHo is used to assess the level of coordination of neural activity in local brain regions by calculating ReHo values to indirectly reflect the spontaneous activity of local neurons in time synchronization (23). ALFF reflects the intensity of spontaneous activity in a region of the brain over a short period of time by calculating the average amplitude of low-frequency oscillations in that region (24). However, the diseases currently studied using a combined ALFF and ReHo approach mainly include Parkinson's disease (26), generalized anxiety disorder (25), bipolar disorder (27), and migraine without aura (28), while differential studies of TRD and nTRD are lacking. So far, there have been some studies on the difference between TRD and nTRD, but most of them use ALFF or ReHo as a single indicator. Previous studies have shown differences in the default mode network (DMN), visual recognition circuits, right auditory, right sensory/somatomotor hand and cerebellar ALFF in the TRD group compared to the nTRD group (29, 30). In addition, other studies have shown differences in ReHo in the left inferior frontal gyrus, right middle temporal gyrus, left precuneus, middle cingulate gyrus and left fusiform gyrus in the TRD group compared to the nTRD group (31, 32). The above rs-fMRI study found that ALFF and ReHo may be highly sensitive to changes in TRD. Previous studies have consistently shown that changes in different indices may represent different sensitivities and that changes in multiple indices in the same brain region during the same period can help improve the sensitivity of the diagnosis (33). In addition, from the results of previous studies, it also can be seen that the main difference between TRD and nTRD is in the frontal and temporal lobes and other brain regions. It has also been shown that patients with TRD have gray matter volume changes in frontal and temporal lobe regions (34). The frontal and temporal lobes are not only important brain regions in the pathogenesis of MDD, but also closely related to the DMN and cognitive control network (CCN) (35–38).

Therefore, we used ALFF and ReHo to differentiate the differences in functional brain activity among the TRD group, nTRD group and healthy control (HC) group based on previous studies, and further analyzed the correlation between the differential brain areas and clinical characteristics. We hypothesized that there are differences in neural circuits in the brain of TRD and nTRD patients, especially closely related to the frontal lobe, temporal lobe. This study will provide a neuroimaging basis for the differences in neuropathological mechanisms between TRD and nTRD, which will also contribute more to the understanding of the pathogenesis of TRD.

## Materials and Methods

### Participants

Seventy-three patients with MDD were from Guang'anmen Hospital, China Academy of Chinese Medical Sciences, Beijing First Hospital of Integrated Chinese and Western Medicine, and Xuanwu Hospital, Capital Medical University. All patients met the MDD criteria of the Diagnostic and Statistical Manual of Mental Disorders, Fifth Edition (DSM-5). We used the 17-item Hamilton Rating Scale for Depression scale (HAMD-17) to assess the severity of depression in all MDD patients and divided these patients into those with TRD (TRD, *n* = 37) and those with nTRD (nTRD, *n* = 36). The inclusion criteria for all patients were: (1) age 18–55 years; (2) HAMD-17 score >17; (3) right-handedness; (4) The TRD group was eligible to receive two or more adequate doses and courses of antidepressant therapy that were not effective and had been stable for more than 6 weeks on antidepressant medication prior to enrollment, with non-responsiveness defined as a decrease in HAMD-17 score of <50%. The nTRD group was in symptomatic remission on one antidepressant, with responsiveness defined as a decrease of more than 50% in HAMD-17 scores, relapse prior to enrollment. Thirty-five gender- and age-matched HCs (21 females and 14 males): (1) age 18–55 years; (2) HAMD-17 score <7; (3) right-handedness; (4) no history of any mental illness in first-degree relatives.

The exclusion criteria for patients and HCs were as follows: (1) history of drug abuse; (2) any contraindications to MRI, such as presence of a heart pacemaker, metal fixed false teeth, or severe claustrophobia; (3) pregnant or lactating status; (4) bipolar disorder, suicidal thoughts, and any other mental illness; (5) tumors, history of head trauma with loss of consciousness, and any cardiovascular, kidney or other major medical condition; (6) previous participation in electrical stimulation therapy.

All patients were required to sign an informed consent form before enrollment. This study was approved by the ethics committee of Guang'anmen Hospital, China Academy of Chinese Medical Sciences.

### Scan Acquisition

All patients in this study underwent MRI using a Magnetom Skyra 3.0-T scanner (Siemens, Erlangen, Germany). Before the scanning procedure, the patients were instructed to remain awake and avoid active thinking. During the scanning process, the patients were required to wear earplugs and noise-canceling headphones, to use a hood to immobilize the head, and to lie flat on the examination bed. The scanning procedure involved a localizer scan, high-resolution three-dimensional T1-weighted imaging, and BOLD-fMRI.

The scanning parameters were as follows: for three-dimensional T1-weighted imaging, time repetition/time echo = 2500/2.98 ms, flip angle = 7°, matrix = 64 × 64, field of view = 256 mm × 256 mm, slice thickness = 1 mm, slice number = 48, slices = 192, scanning time = 6 min 3 s; for BOLD-fMRI, time repetition/time echo = 2000/30 ms, flip angle = 90°, matrix = 64 × 64, field of view = 240 mm × 240 mm, slice number = 43, slice thickness/spacing = 3.0/1.0 mm, number of obtained volumes = 200, and scanning time = 6 min 40 s.

### Image Processing

#### fMRI Data Preprocessing

The acquired rs-fMRI data were pre-processed using MATLAB-based DPARSF 5.1 software (DPARSF 5.1, http://www.rfmri.org/DPARSF). (1) conversion of DICOM raw data to NIFTI format; (2) removal of the first 10 time points in order to put the data in a stable state; (3) slice-timing; (4) realignment of head-motion (removal of patients with head movements > 2 mm in any direction and motor rotation >2°); (5) regression of covariates, including brain white matter signal, cerebrospinal fluid signal and head movement parameters; (6) spatial normalization: functional images of all subjects were converted to Montreal Neurological Institute (MNI) standard space using DARTEL; (7) linear detrending and filtering (0.01 to 0.08 Hz).

#### ALFF Analysis

The data were spatially normalized and smoothed, and then a fast fourier transform (FFT) was performed to switch the time series to the frequency domain to obtain the power spectrum. The square root of the power spectrum at each frequency is calculated to obtain the average square root of the ALFF measurement for each voxel in the range of 0.01 to 0.08 Hz. Finally, time bandpass filtering (0.01 to 0.08 Hz) is then performed. To reduce inter-individual variability, ALFF was transformed to zALFF by Fisher's z transformation prior to statistical analysis.

#### ReHo Analysis

The similarity of the time series of each voxel to its neighboring voxels (26 neighboring voxels) was assessed using the Kendall's coefficient concordance(KCC), i.e., ReHo values. The whole-brain ReHo images of the subjects were obtained by calculating the KCC values of the whole-brain voxels. To improve the signal-to-noise ratio, the ReHo images were spatially smoothed using a 6 mm × 6 mm × 6 mm full-width half-height Gaussian kernel.

### Statistical Analyses

#### Clinical Data Analysis

Clinical data were analyzed with SPSS 23.0 statistical software (IBM Corporation, Somers, New York). One-way analysis of variance (ANOVA) was used to compare ages and education levels among the three groups, and the chi-square test was used to compare gender. A two-sample *t*-test was used to compare the duration of disease and HAMD-17 scores between the two groups, with *P* < 0.05 (two-tailed) as the threshold for statistical significance.

#### fMRI Data Analysis

Image data were analyzed using the DPARSF toolbox, and a voxel-based one-way ANOVA was performed to compare whole-brain ALFF/ReHo map of the three groups. Gender, age, education level and framewise displacement (FD) metric (derived from Jenkinson's formula) of the three groups of subjects were used as covariates, and brain areas with ALFF/ReHo differences between the three groups were corrected for Gaussian random fields (GRF), and corrected cluster levels were set as *P* < 0.05 (two-tailed) and threshold voxel levels *P* < 0.005 were defined as statistically different. We performed *post-hoc t*-test analysis using DPARSF 5.1 software for two-by-two comparisons between groups, and Bonferroni correction was applied to the results, setting a threshold of *P* < 0.016 (0.05/3) for statistical significance.

In order to verify the relationship between ALFF/ReHo values and clinical symptoms, we extracted the mean ALFF/ReHo values of the three different brain regions and did Pearson correlation analysis with the clinical scale scores of each group, and the statistical threshold of *P* < 0.05 (two-tailed) was statistically significant.

## Results

### Characteristics of Research Samples

Two patients with TRD and one patient with nTRD were excluded because of excessive head movement. Therebefore, a total of 35 patients with TRD, 35 patients with nTRD, and 35 HCs were eligible for the study criteria. There were no statistical differences among the three groups in terms of gender, age and years of education. There was no statistical difference between the TRD and nTRD groups in terms of HAMD-17 scores and a statistical difference in terms of illness duration **(****Table 1****)**.

**Table 1:** Demographic and clinical characteristics of the study participants.

**Variable**	**TRD group (*n*, 35)**	**nTRD group (*n*, 35)**	**HC group(*n*, 35)**	***t(F)/χ*2**	***P*-value**
Gender (M/F)	13/22	15/20	14/21	0.238	0.888^a^
Age (years)	36.65 ± 9.63	37.14 ± 9.85	37.48 ± 10.82	0.059	0.943^b^
Education (years)	14.20 ± 3.07	14.48 ± 2.70	14.37 ± 3.56	0.074	0.929^b^
Duration of illness (months)	48.45 ± 18.75	23.57 ± 13.99	NA	6.292	<0.001^c*^
HAMD-17 score	23.28 ± 3.73	22.57 ± 3.55	NA	0.820	0.415^c^

*TRD, treatment resistant depression; nTRD, non-treatment-resistant depression; HC, healthy control; HAMD-17, 17-item Hamilton Rating Scale for Depression; NA; not applicable*.

*^a^The P-values of gender distribution among the three groups were obtained by chi-square test.*

*^b^The P-values were obtained by one-way analysis of variance tests.*

*^c^The P-value obtained by two-sample t-test.*

*Significant difference.

### Differences in ALFF/ ReHo Among the TRD Group, nTRD Group, and HC Group

**Figure 1:**
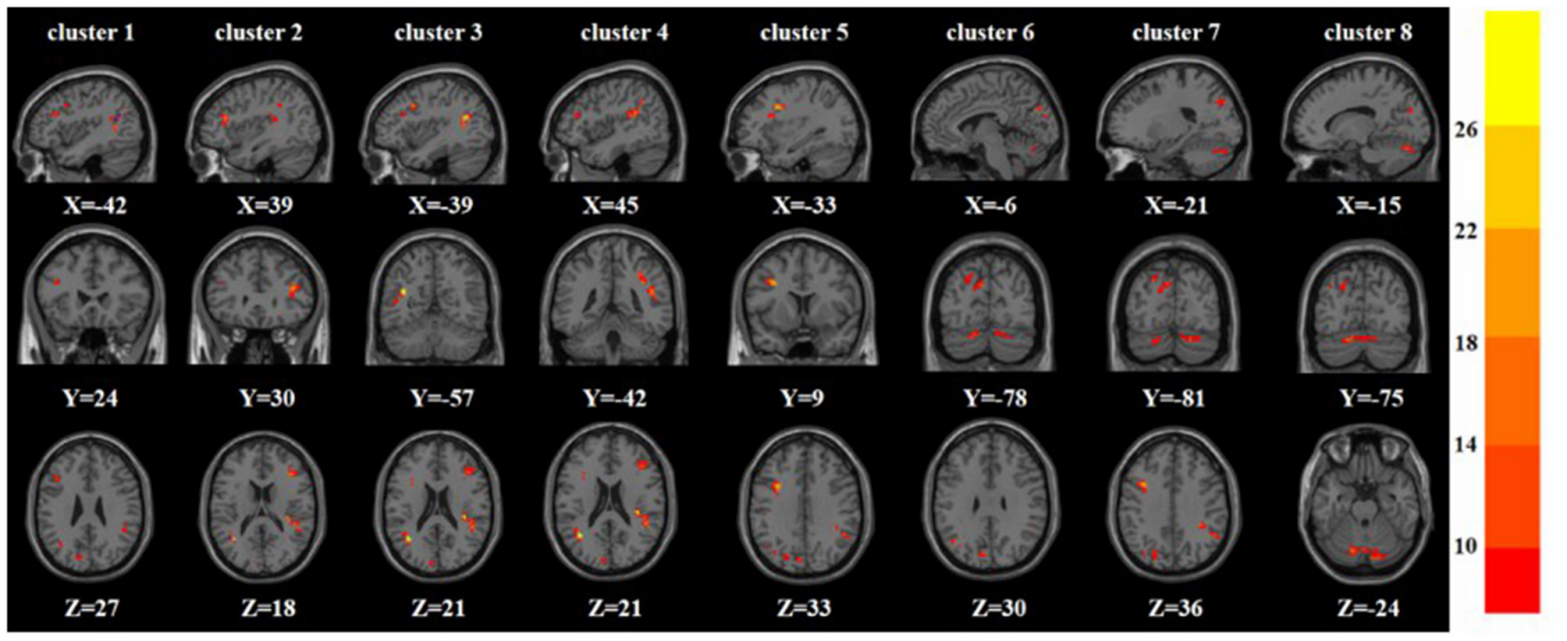
Based on one-way ANOVA for three groups of ALFF abnormal brain regions. The color bars indicate the *F*-value.

**Figure 2:**
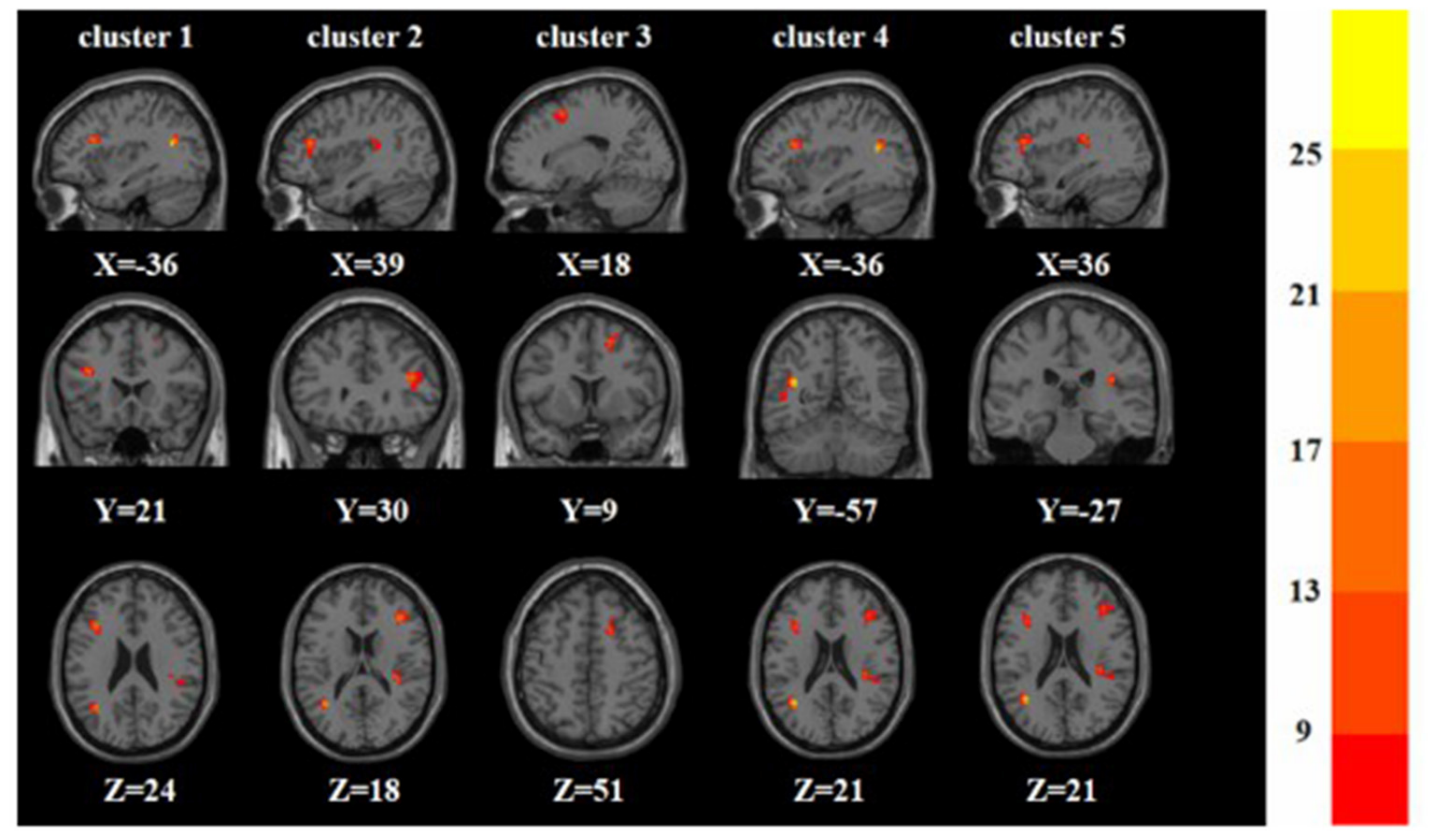
Based on one-way ANOVA for three groups of ReHo abnormal brain regions. The color bars indicate the *F*-value.

A one-way ANOVA revealed significant differences in ALFF and ReHo among the three groups in the left/right inferior frontal triangular gyrus, left middle temporal gyrus and right superior temporal gyrus. Meanwhile, there were significant differences among the three groups in ALFF in the left precentral gyrus, left cuneus, left superior occipital gyrus, and bilateral posterior lobes of the cerebellum, and significant differences in ReHo in the right superior frontal gyrus (Tables 2, 3 and Figures 1, 2).

**Table 2:** ALFF differences in TRD group; nTRD group; and HC group.

**Clusters**	**Brain regions**	**MNI Peak**			**Cluster size**	***F/T*–value (peak)**
		**X**	**Y**	**Z**		
**Differences among three groups**
1	Left inferior frontal triangular gyrus	−42	24	27	19	12.195
2	Right inferior frontal triangular gyrus	39	30	18	44	19.130
3	Left middle temporal gyrus	−39	−57	21	67	28.318
4	Right superior temporal gyrus	45	−42	21	60	17.731
5	Left precental gyrus	−33	9	33	34	23.937
6	Left cuneus	−6	−78	30	29	10.982
7	Left superior occipital gyrus	−21	−81	36	21	10.729
8	Bilateral posterior lobes of the cerebellum	−15	−75	−24	102	15.192
**TRD group vs. nTRD group**
1	Left inferior frontal triangular gyrus	−45	27	27	18	−2.914
2	Right inferior frontal triangular gyrus	36	33	18	35	−2.837
3	Left middle frontal gyrus	−36	12	36	31	5.129
4	Left middle temporal gyrus	−42	−54	9	14	−2.932
5	Right superior temporal gyrus	45	−42	21	23	5.108
6	Left cuneus	−15	−81	24	24	−2.813
7	Bilateral posterior lobes of the cerebellum	15	−72	−24	87	−2.813
**TRD group vs. HC group**
1	Right inferior frontal triangular gyrus	45	30	21	18	−2.850
2	Left middle frontal gyrus	−33	9	33	25	5.529
3	Left middle temporal gyrus	−48	−57	9	40	−2.831
**nTRD group vs. HC group**
1	Left angular gyrus	−39	−60	27	27	−2.808
2	Right superior temporal gyrus	45	−36	21	13	3.839

*MNI Peak; Coordinates of primary peak locations in the Montreal Neurological Institute space*.

**Table 3:** ReHo differences in TRD group; nTRD group; and HC group.

**Clusters**	**Brain regions**	**MNI Peak**			**Cluster size**	***F/T*–value (peak)**
		**X**	**Y**	**Z**		
**Differences among three groups**
1	Left inferior frontal triangular gyrus	−36	21	24	32	15.834
2	Right inferior frontal triangular gyrus	39	30	18	53	16.467
3	Right superior frontal gyrus	18	9	51	40	13.174
4	Left middle temporal gyrus	−36	−57	21	60	27.620
5	Right superior temporal gyrus	36	−27	21	42	13.949
**TRD group vs. nTRD group**
1	Left inferior frontal triangular gyrus	−39	24	21	30	−2.944
2	Right inferior frontal triangular gyrus	42	30	18	29	−2.878
3	Right superior frontal gyrus	18	9	54	22	4.317
4	Left middle temporal gyrus	−45	−57	6	20	−2.833
**TRD group vs. HC group**
1	Right inferior frontal triangular gyrus	42	33	15	27	−2.818
2	Left middle temporal gyrus	−45	−54	3	35	−2.834
**nTRD group vs. HC group**
1	Left angular gyrus	−36	−63	21	27	−2.847
2	Right insula	36	−27	21	20	3.038

*MNI Peak; Coordinates of primary peak locations in the Montreal Neurological Institute space*.

**Figure 3:**
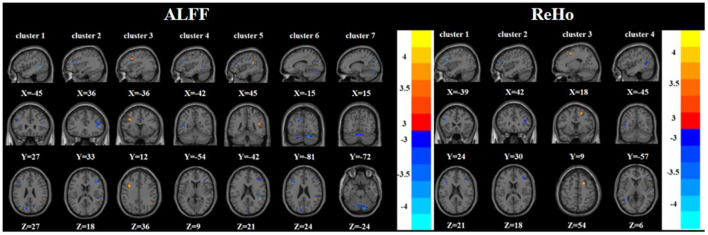
Brain regions with abnormal ALFF (left) and ReHo (right) between TRD group and nTRD group based on *post hoc T*-tests. The color bars indicate the *T*-value.

Compared with the nTRD group, the TRD group had increased ALFF in the left middle frontal gyrus, right superior temporal gyrus, and decreased ALFF in the left/right inferior frontal triangular gyrus, left middle temporal gyrus, left cuneus, and bilateral posterior lobes of the cerebellum. On the other hand, the TRD group had increased ReHo in the right superior frontal gyrus and decreased ReHo in the left/right inferior frontal triangular gyrus, and left middle temporal gyrus (Tables 2, 3 and Figure 3).

**Figure 4:**
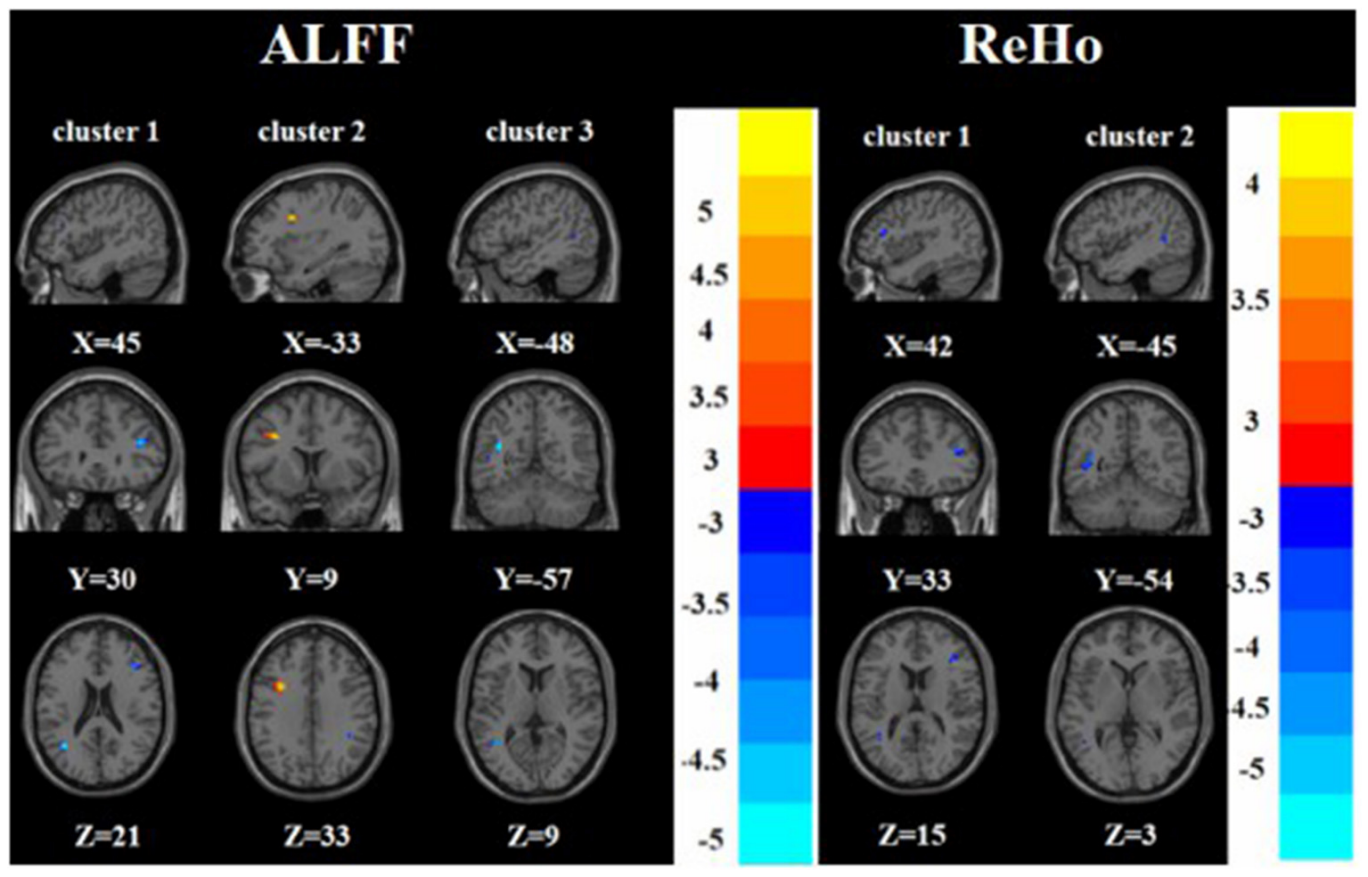
Brain regions with abnormal ALFF (left) and ReHo (right) between TRD group and HC group based on *post hoc T*-tests. The color bars indicate the *T*-value.

Compared with the HC group, the TRD group had increased ALFF in the left middle frontal gyrus and decreased ALFF in the right inferior frontal triangular gyrus and left middle temporal gyrus. On the other hand, the TRD group had decreased ReHo in the right inferior frontal triangular gyrus and left middle temporal gyrus (Tables 2, 3 and Figure 4).

**Figure 5:**
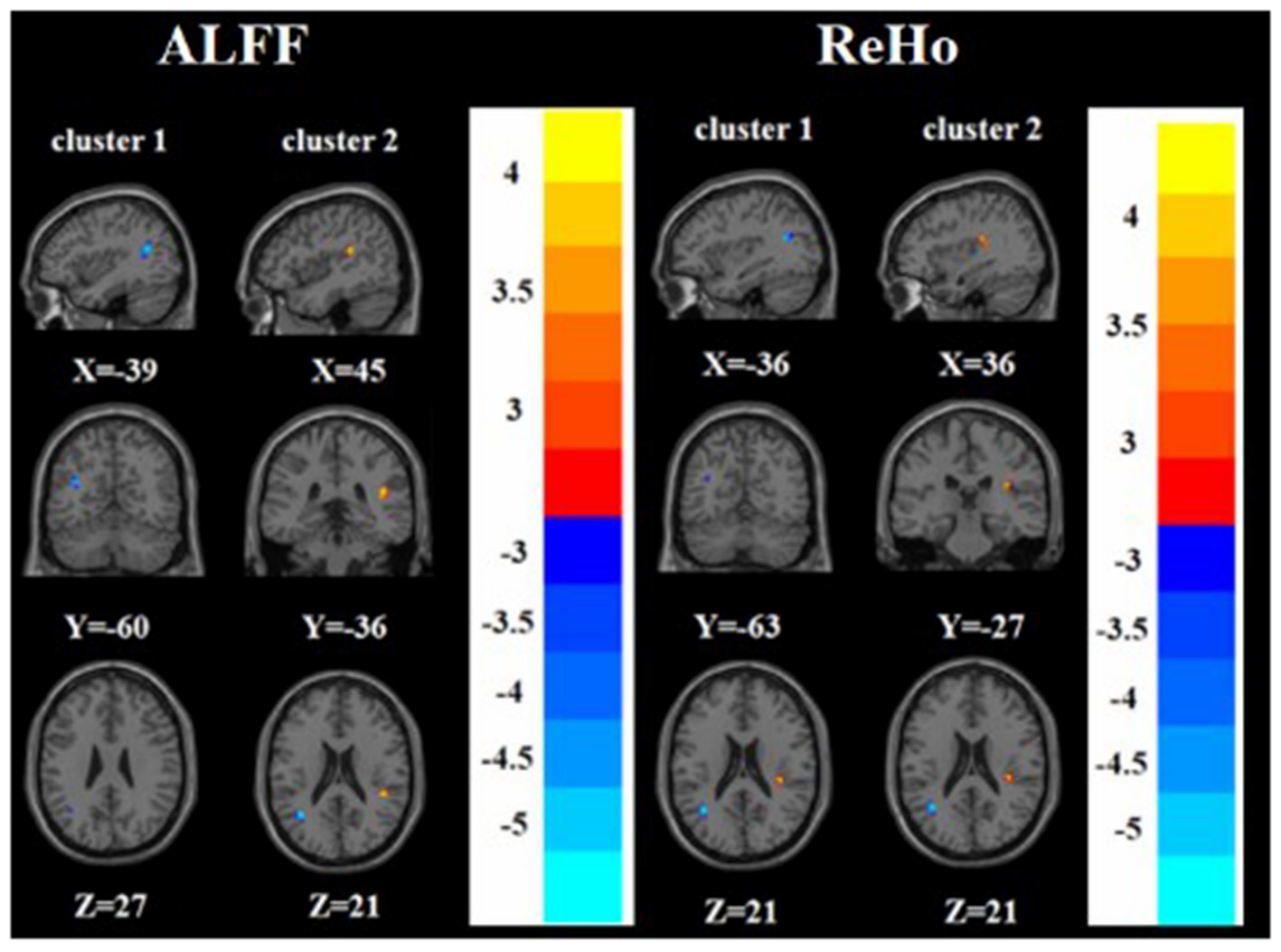
Brain regions with abnormal ALFF (left) and ReHo (right) between nTRD group and HC group based on *post hoc T*-tests. The color bars indicate the *T*-value.

Compared with the HC group, the nTRD group had increased ALFF in the right superior temporal gyrus and decreased ALFF in the left angular gyrus. On the other hand, the nTRD group had increased ReHo in the right insula and decreased ReHo in the left angular gyrus (Tables 2, 3 and Figure 5).

### Significant Correlation Between Functional Image and Clinical Feature

**Figure 6:**
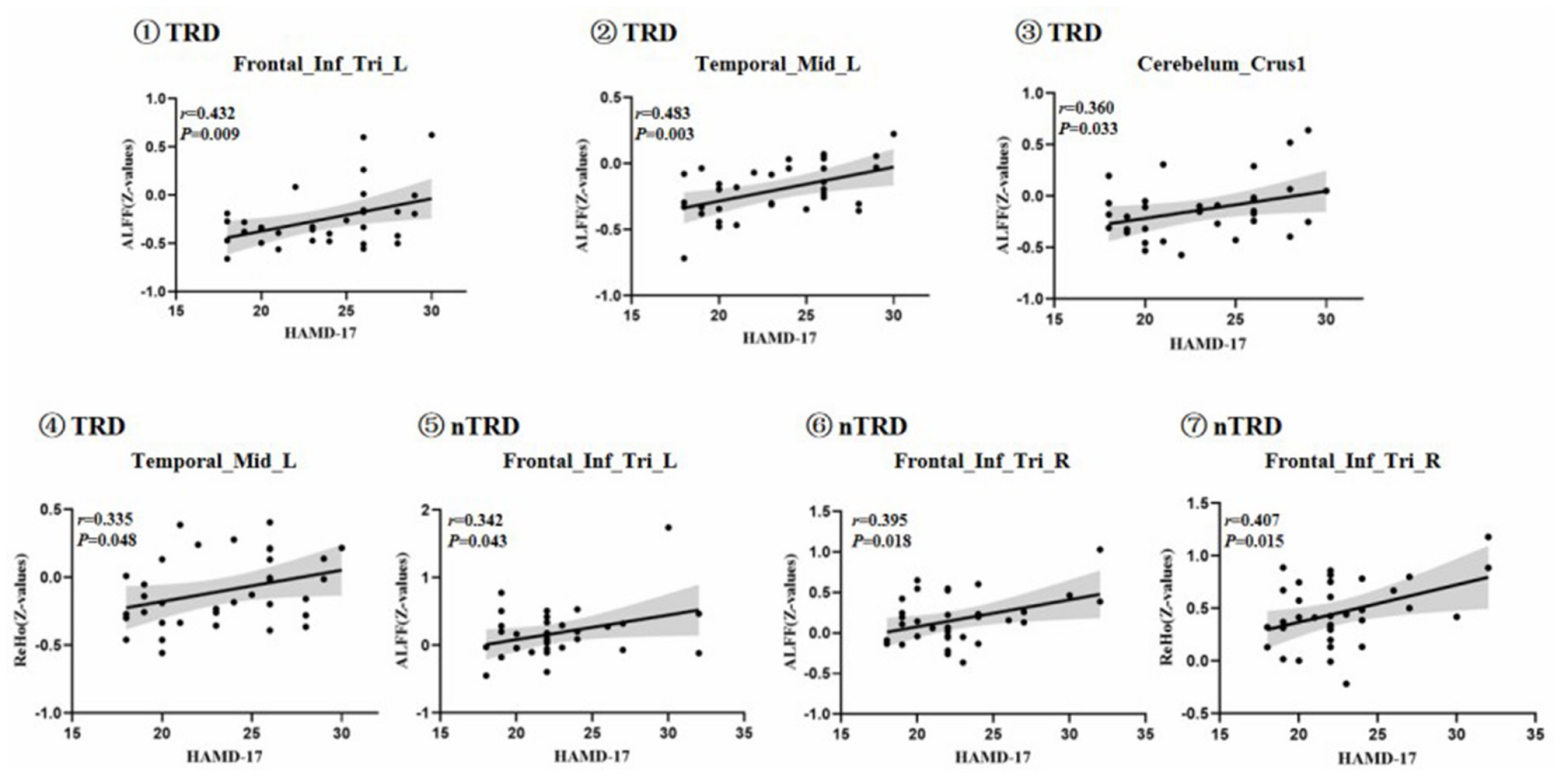
Positive correlation between the ALFF and ReHo of the abnormal brain regions: ①Frontal_Inf_Tri_L, Left inferior frontal triangular gyrus; ALFF value of TRD group; ②Temporal_Mid_L, left middle temporal gyrus; ALFF value of TRD group; ③Cerebelum_Crus1, bilateral posterior lobes of the cerebellum(Crus I); ALFF value of TRD group; ④Temporal_Mid_L, left middle temporal gyrus; ReHo value of TRD group; ⑤Frontal_Inf_Tri_L, Left inferior frontal triangular gyrus; ALFF value of nTRD group; ⑥Frontal_Inf_Tri_R, Right inferior frontal triangular gyrus; ALFF value of nTRD group; ⑦Frontal_Inf_Tri_R, Right inferior frontal triangular gyrus; ReHo value of nTRD group.

To test the correlation between areas of abnormal brain activity and the severity of clinical depressive symptoms, we further performed Pearson correlation analysis. We found that the ALFF values of the left inferior frontal triangular gyrus, left middle temporal gyrus, and bilateral posterior lobes of the cerebellum in the TRD group were positively correlated with the HAMD-17 score (*r* = 0.432, *P* = 0.009; *r* = 0.483, *P* = 0.003; *r* = 0.360, *P* = 0.033). Meanwhile, the ReHo values of the left middle temporal gyrus in the TRD group were positively correlated with the HAMD-17 scores (*r* = 0.335, *P* = 0.048). On the other hand, the ALFF values of the left/right inferior frontal triangular gyrus in the nTRD group were positively correlated with the HAMD-17 score (*r* = 0.342, *P* = 0.043; *r* = 0.395, *P* = 0.018). In addition, the ReHo values of the right inferior frontal triangular gyrus in the nTRD group were positively correlated with the HAMD-17 score (*r* = 0.407, *P* = 0.015) (Table 4 and Figure 6).

**Table 4:** Correlation of abnormal brain areas with clinical symptoms.

**Variables**	**Group**	**Brain regions**	**HAMD−17 score**	
			**Coefficient**	***P*–value**
ALFF	TRD	Left inferior frontal triangular gyrus	0.432	0.009^d#^
		Left middle temporal gyrus	0.483	0.003^d#^
		Bilateral posterior lobes of the cerebellum	0.360	0.033^d#^
	nTRD	Left inferior frontal triangular gyrus	0.342	0.043^d#^
		Right inferior frontal triangular gyrus	0.395	0.018^d#^
ReHo	TRD	Left middle temporal gyrus	0.335	0.048^d#^
	nTRD	Right inferior frontal triangular gyrus	0.407	0.015^d#^

^d^*P–value for pearson correlation(not corrected)*.

^#^*Statistical significance*.

## Discussion

To our knowledge, this is the first study of the relationship between changes in local functional brain activity in TRD and nTRD using ALFF and ReHo. The results of this study showed no significant differences in clinical depressive symptoms between the TRD and nTRD groups, but abnormal neurofunctional activity in some of the same brain regions. Compared with the nTRD group, the brain regions of ALFF and ReHo in the TRD group were more extensively altered, and the neuropathological mechanisms was more complex. This study provides a reference value for the differences in functional brain activity between TRD and nTRD.

In this study, we found TRD group had decreased ALFF/ReHo in the left/right inferior frontal triangular gyrus compared to the nTRD group. The inferior frontal triangular gyrus is located in the pre-frontal lobe, near the orbito frontal cortex (OFC) and the middle frontal gyrus, and is a key brain region for emotional and cognitive control circuits, and is associated with certain types of behavioral inhibition (39–41). The inferior frontal triangular gyrus is not only an important part of the CCN, but is also closely related to MDD (38, 39). In addition, the inferior frontal triangle gyrus is known for its involvement in speech function and language processing (42). Patients with MDD are characterized by a wide range of abnormalities in language comprehension (43). A study suggests that glutamatergic neurotransmission may play a regulatory role in language acquisition, comprehension, and production (44). Abnormal glutamate signaling plays an important role in the etiology of MDD (45, 46). More research is needed to understand how MDD-related genes affect the inferior frontal triangular gyrus. Previous studies have also found ALFF differences in the TRD group, treatment response depression (TSD) group and HC group in the inferior frontal gyrus (32). Another study found that TRD had decreased ReHo in the left inferior frontal gyrus than in the nTRD group, which is consistent with the results of this study (31). A study also found that significantly dereduced glucose metabolism in the right inferior frontal gyrus of patients with MDD was associated with the severity of pleasure deficits in depression (47). Therefore, the results of this study showed that ALFF and ReHo were decreased in the TRD group compared to the nTRD group, suggesting a more severely impaired inferior frontal triangular gyrus in the TRD group. Correlation analysis showed that ALFF/ReHo values in the left/right inferior frontal triangular gyrus of the nTRD group were positively correlated with HAMD-17 scores, suggesting that the occurrence of depressive symptoms in the nTRD group was more closely related to dysfunction in the inferior frontal triangular gyrus, and that the inferior frontal triangular gyrus was an important differential brain area between the TRD and nTRD groups.

We found that the TRD group had increased ALFF in the left middle frontal gyrus and ReHo in the right superior frontal gyrus compared to the nTRD group. The left middle frontal gyrus and the right superior frontal gyrus are key brain regions of the dorsolateral prefrontal cortex (DLPFC) and an important component of the CCN (48, 49). Several studies have shown that treatment with repetitive transcranial magnetic stimulation and transcranial direct current stimulation in the CCN, especially DLPFC, can directly improve core symptoms such as mood and cognition in MDD patients (50). Previous studies also found that the TRD group had significantly increased ALFF in the left DLPFC compared to the nTRD group, which is consistent with the results of the present study (30). Therefore, the results of this study suggest that both ALFF and ReHo are sensitive to the left middle frontal gyrus in TRD patients, suggesting that impaired function of the DLPFC is one of the causes of the complex pathological mechanisms in TRD patients.

We also found that ALFF/ReHo in the left middle temporal gyrus was decreased in the TRD group compared to the nTRD group. The middle temporal gyrus is involved in tasks related to lexical cognition and semantic understanding in humans and is important for understanding visual and auditory information (51, 52). The possibility of functional disruption of the middle temporal gyrus in MDD and schizophrenia exists (53, 54). The middle temporal gyrus is also closely related to the DMN and is the basis of the DMN in the functional role of language (51). Previous studies have also found differences in ReHo in the right middle temporal gyrus between the TRD and nTRD groups (31). A clinical study also showed that electroconvulsive therapy improved TRD and was associated with the ability to enhance high connectivity between the anterior subgenual anterior cingulate and the middle temporal gyrus (55). Therefore, both ALFF and ReHo in the left middle temporal gyrus were decreased in the TRD group in this study, suggesting that compensatory hypofunction of the left middle temporal gyrus may be an important mechanism in the pathogenesis of TRD patients. In addition, correlation analysis showed that ALFF and ReHo values of the left middle temporal gyrus in the TRD group were positively correlated with HAMD-17 scores, while this was not found in the nTRD group, suggesting that the left middle temporal gyrus may be a neuroimaging marker of TRD.

The right superior temporal gyrus also plays an important role in social-emotional processing as part of the DMN (56, 57). Patients with TRD are vulnerable to suicide risk during adolescence, and reduced volume of the right superior temporal gyrus can be a marker for suicide attempts during adolescence (57). Previous studies have found ReHo was decreased in the right superior temporal gyrus in TRD group than in the HC group (31). The results of this study showed that ALFF in the right superior temporal gyrus was increased in the TRD group compared to the nTRD group, indicating an important differential brain area between TRD and nTRD in the right superior temporal gyrus.

In this study, ALFF in the left cuneus was decreased in the TRD group compared to the nTRD group. The cuneus is part of the occipital lobe and is involved in visual perception functions (e.g., facial emotion) and plays an important role in social interaction (58, 59). Functional changes in the cuneus lobe are also closely related to MDD (60, 61). A study of an fMRI reward processing task found that adolescents with unremitting depression showed greater activation in the frontal middle gyrus and less activation in the cuneus compared to adolescents with remitting depression (62). Previous studies found that the ReHo in the left precuneus was decreased in the TRD group compared to the nTRD group (31). Another study also found decreased FC between the medial pre-frontal cortex and the cuneus in the TRD group compared to the nTRD group (63). Therefore, the results of this study suggest that abnormal function of the left cuneus is one of the reasons for the complex neuropathological mechanism of TRD.

Compared with the nTRD group, the ALFF of bilateral posterior lobes of the cerebellum in the TRD group was decreased compared to the nTRD group. In addition to the motor domain, the cerebellum is also involved in the cognitive and emotional aspects of the human body (64). Atrophy of the cerebellum often leads to cognitive and emotional symptoms, sometimes referred to as “cerebellar cognitive-emotional syndrome” (65). Cerebellar damage predisposes to language processing, aspects of executive function, and emotional dysregulation (66). Animal model studies have shown that electrical stimulation experiments also link cerebellar neural activity to depression and impulsive behavior (67). Previous studies have shown that ReHo was decreased in the bilateral cerebellum in the TRD group compared to the TSD group. Therefore, the results of the present study suggest that the posterior lobes of the cerebellum is further involved in emotion regulation in patients with TRD. In addition, correlation analysis showed a positive correlation between ALFF values and HAMD-17 scores in the bilateral posterior lobes of the cerebellum. Therefore, bilateral posterior lobes of the cerebellum may be a neuroimaging marker in patients with TRD.

Meanwhile, we also found that the TRD group had decreased ALFF/ReHo in the right inferior frontal triangular gyrus and left middle temporal gyrus compared to the HC group. Previous studies found that the TRD group had decreased ALFF in the right inferior frontal gyrus and left middle temporal gyrus compared to the HC group, which is consistent with the present study (68). However, it has also been found that the ReHo in the TRD group was decreased in the left lateral inferior frontal gyrus than in the HC group, and increased in the right middle temporal gyru scompared to the HC group, which is different from the present study (31). This variation in results may be related to differences in patient medication use, sample size and scanning and analysis methods. However, all of the above studies suggest that the inferior frontal gyrus and middle temporal gyrus are important differential brain regions between the TRD and HC groups. Interestingly, the inferior frontal triangular gyrus and middle temporal gyrus were also important differences between the TRD and nTRD groups, while no differences were found between the nTRD and HC groups in these two brain regions, therefore, further attention and research on these two brain regions are needed in the future.

Some limitations should be considered. First, although the patients in this study were included strictly according to the inclusion criteria, there were still some factors that potentially influenced the results of this study, such as the time of onset, the use of antidepressants and the duration of the disease. Second, this study focused on only one scale, the HAMD-17, and more scales need to be used in the future to focus in more detail on the correlation between cognitive, anxiety, insomnia, and somatic subtypes of symptoms with TRD and nTRD in patients with MDD, thus improving the scientific value of this study. Third, we did not clarify the gender differences between the different groups of the sample in this study, so this is an important direction for future research. Finally, Setting a GRF correction threshold voxel level of *P* < 0.001 is more statistically valid (69, 70), but we did not find significant cluster. Setting a threshold of *P* < 0.005 in this study may be slightly statistically weak, and in future studies, we will further expand the sample size and use more stringent statistical validity to enhance the scientific value of this study.

## Conclusion

In conclusion, we used ALFF and ReHo based on rs-fMRI technique to preliminarily analyze the differences in local neurological functional activity between TRD and nTRD brains. We found that although the clinical symptoms were similar in the TRD and nTRD groups, there was abnormal neurofunctional activity in some of the same brain regions, and ALFF and ReHo were more extensively altered in the TRD group with more complex neuropathological mechanisms, especially in the inferior frontal triangular gyrus of the frontal lobe and the middle temporal gyrus of the temporal lobe.

## Data Availability Statement

The raw data supporting the conclusions of this article will be made available by the authors, without undue reservation.

## Ethics Statement

The studies involving human participants were reviewed and approved by the Ethics Committee of Guang'anmen Hospital, Chinese Academy of Traditional Chinese Medicine (NO. 2017-021-SQ). The patients/participants provided their written informed consent to participate in this study.

## Author Contributions

JF conceived and designed this experiment. JS drafted the manuscript and participated in data collection and analysis. YM drew diagrams and made statistical analysis of data. LC, ZW, CG, YL, DG, XL, and KX involved in data analysis and data collection. YH performed fMRI on the subjects. XH, JT, XY, HW, and XX involved in case collection and symptom assessment of patients. All authors contributed to the article and approved the submitted version.

## Funding

This research was supported by the China Academy of Chinese Medical Sciences Innovation Fund (CI2021A03301), National Natural Science Foundation of China (82174282 and 81774433), National Key Research and Development Program of China (2018YFC1705802), and Clinical Efficacy and Brain Mechanism of Transcutaneous Auricular Vagus Nerve Stimulation for patients With Mild to Moderate Depression (QYSF-2020-02).

## Conflict of Interest

The authors declare that the research was conducted in the absence of any commercial or financial relationships that could be construed as a potential conflict of interest.

## Publisher's Note

All claims expressed in this article are solely those of the authors and do not necessarily represent those of their affiliated organizations, or those of the publisher, the editors and the reviewers. Any product that may be evaluated in this article, or claim that may be made by its manufacturer, is not guaranteed or endorsed by the publisher.
